# Bioinformatics-Driven Multi-Factorial Insight into α-Galactosidase Mutations

**DOI:** 10.3390/ijms26125802

**Published:** 2025-06-17

**Authors:** Bruno Hay Mele, Federica Rossetti, Giuseppina Andreotti, Maria Vittoria Cubellis, Simone Guerriero, Maria Monticelli

**Affiliations:** 1 Department of Biology, University of Napoli “Federico II”, 80126 Napoli, Italysim.guerriero@studenti.unina.it (S.G.);; 2 Institute of Biomolecular Chemistry (ICB)—National Council Research of Italy, 80078 Pozzuoli, Italy

**Keywords:** AGAL, Fabry disease, missense mutations, structural bioinformatics, AlphaMissense, EVE, FoldX, ChimeraX

## Abstract

Fabry disease is a rare genetic disorder caused by deficient activity of the lysosomal enzyme alpha-galactosidase A (AGAL), resulting in the accumulation of globotriaosylceramides (Gb3) in tissues and organs. This buildup leads to progressive, multi-systemic complications that severely impact quality of life and can be life-threatening. Interpreting the functional consequences of missense variants in the *GLA* gene remains a significant challenge, especially in rare diseases where experimental evidence is scarce. In this study, we present an integrative computational framework that combines structural, interaction, pathogenicity, and stability data from both in silico tools and experimental sources, enriched through expert curation and structural analysis. Given the clinical relevance of pharmacological chaperones in Fabry disease, we focus in particular on the structural characteristics of variants classified as “amenable” to such treatments. Our multidimensional analysis—using tools such as AlphaMissense, EVE, FoldX, and ChimeraX—identifies key molecular features that distinguish amenable from non-amenable variants. We find that amenable mutations tend to preserve protein stability, while non-amenable ones are associated with structural destabilisation. By comparing AlphaMissense with alternative predictors rooted in evolutionary (EVE) and thermodynamic (FoldX) models, we explore the relative contribution of different biological paradigms to variant classification. Additionally, the investigation of outlier variants—where AlphaMissense predictions diverge from clinical annotations—highlights candidates for further experimental validation. These findings demonstrate how combining structural bioinformatics with machine learning–based predictions can improve missense variant interpretation and support precision medicine in rare genetic disorders.

## 1. Introduction

Fabry disease (FD; OMIM #301500, ORPHANET 324) is a rare X-linked lysosomal storage disorder caused by deficient activity of the enzyme α-galactosidase A (AGAL; EC 3.2.1.22) [1,2,3]. Loss or reduction in AGAL enzymatic function leads to pathological accumulation of globotriaosylceramide (Gb3) and related glycosphingolipids in the lysosomes of vascular endothelial, renal, cardiac, and neuronal cells. This accumulation progressively disrupts cellular function, giving rise to the diverse clinical manifestations of the disease [4].

The molecular basis of FD is highly heterogeneous, with more than a thousand distinct mutations reported in the *GLA* gene [5]. These include splicing variants, nonsense mutations, frameshift insertions or deletions, and missense substitutions [6]. The latter are thought to affect protein folding, structural stability, catalytic efficiency, or protein–protein/cofactor interactions, depending on their position. Variants occurring in the active site often abolish enzymatic activity and are associated with severe phenotypes. In contrast, mutations located away from the active site—such as those at the dimerisation interface—can compromise protein stability or trafficking and are typically associated with milder forms of the disease [7].

Among the most widely adopted treatment strategies for FD are enzyme replacement therapies (ERT) [8] and pharmacological chaperones (PCT) [9], both aiming to reduce the intracellular Gb3 burden. Migalastat, a small-molecule chaperone approved for amenable mutations, binds and stabilises specific mutant AGAL proteins, promoting their proper trafficking to lysosomes [10]. Recent research suggests that small molecules chosen among approved drugs or nutraceuticals can potentiate the effect of pharmacological chaperones [11,12,13]. However, the efficacy of chaperone therapy is highly dependent on the mutation [14], and several attempts have been carried out to try and predict which AGAL mutations could respond to the treatment with pharmacological chaperones [15,16,17].

Recent advances in computational biology and artificial intelligence have enabled the development of machine learning (ML)-based tools for variant effect prediction. These models, trained on large biological datasets, can infer complex relationships between sequence, structure, and function, offering valuable insights into the pathogenic potential of genetic variants. Among these, transformers and variational autoencoders (VAE) have emerged as compelling approaches, capable of generating informative internal representations (“embeddings”) of protein sequences and structure.

VAEs are generative models that learn to represent complex data, such as protein sequences, in a lower-dimensional latent space while preserving key features of their variability. By modelling the probability distribution of sequences, VAEs can capture the underlying constraints shaped by evolution, enabling unsupervised inference of how mutations may alter protein function [18]. In contrast, transformers are attention-based models able to process sequences in parallel (meaning they consider all the residues of the sequence at the same time) and learn context-dependent relationships between residues across long ranges [19]. Both approaches offer powerful frameworks for predicting the effects of genetic variation by learning directly from sequence data.

Two notable ML-based predictors are AlphaMissense [20] and EVE (Evolutionary Variation Effect) [21]. AlphaMissense is based on the transformer paradigm and integrates sequence and structural features to assess the pathogenicity of missense variants. EVE uses instead a VAE architecture to quantify the deleteriousness of variants using evolutionary constraints inferred from large-scale multiple sequence alignments. These tools offer complementary perspectives on variant interpretation—one rooted in molecular structure, the other in evolutionary conservation—and are particularly promising for understudied or rare diseases with high mutational heterogeneity, such as Fabry disease. A recent review underscored the growing relevance of AI applications in rare diseases, highlighting Fabry disease as a model case for integrating computational insights with clinical and biological data [22].

In this study, we built an annotated AGAL variant catalogue and used it as a model to investigate the capabilities of modern computational tools in variant interpretation. We systematically characterise these variants using AlphaMissense and EVE to assess their effectiveness in capturing biologically meaningful signals relevant to disease. By integrating these predictions with complementary data on structural stability (FoldX), molecular dynamics (RMSF), and variant annotations, we evaluate how machine learning models reflect key structural and functional properties of AGAL and their potential to inform therapeutic strategies, including responsiveness to pharmacological chaperones.

## 2. Results and Discussion

### 2.1. Qualitative Assessment of AlphaMissense Performance on AGAL

**Figure 1:**
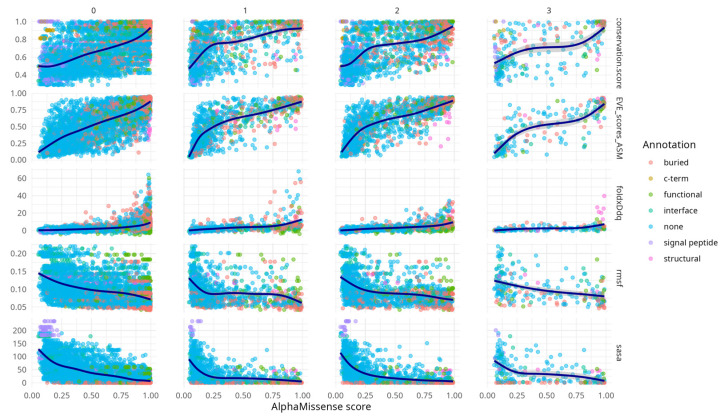
Scatter Plot Matrix displaying the relationships between multiple variables while stratifying them by classes—specifically, the position that the mutation has in the codon. The rows of the matrix show how the AlphaMissense score varies with respect to other indicators (SASA, RMSF, conservation, EVE score, and FoldX score), while the columns divide the plots by codon position (where 0 indicates mutations that cannot be achieved by a single nucleotide substitution). The colour coding represents protein annotations. Detailed analysis for specific cases in Figure 4, Figure 5 and Figure 7.

An exploratory analysis of various aggregated metrics, stratified by codon position, provided qualitative insights into the performance of AlphaMissense (Figure 1). 60% of all possible mutations were clustered in the first column (0), while the fewest (3%) appeared in the fourth (3), reflecting the fact that many substitutions are not achievable via a single nucleotide change—the first column (0) included substitutions not achievable via SNP. The limited number of amino acid variants (250) in the third codon position is consistent with the predominance of synonymous mutations at that site.

Spearman correlation analysis between metrics (Table A1) confirms widespread correlation across the metrics (minimum |rho| > 0.44) with peak correlation between AlphaMissense score and EVE score (rho = 0.8, CI [0.79, 0.82]) and between EVE and conservation (rho = 0.75, CI [0.74, 0.77]). From the second codon position onwards, correlations between AlphaMissense scores and associated metrics became evident. Notably, conservation scores, FoldX ΔΔG values, and EVE predictions displayed concordant increasing trends with AlphaMissense scores. This behaviour is expected, particularly between EVE and conservation, given that EVE is trained on multiple sequence alignments that inherently capture evolutionary conservation.

**Figure A1:**
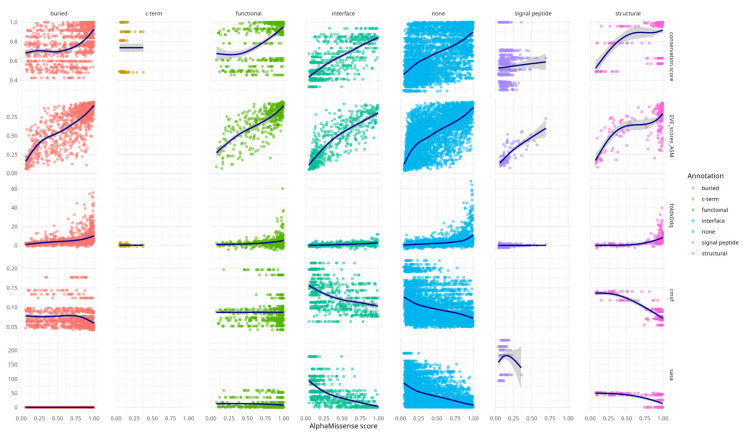
Scatter Plot Matrix displaying the relationships between multiple variables while stratifying them by structural annotation. The rows of the matrix show how the AlphaMissense score varies with respect to other indicators (SASA, RMSF, conservation, EVE score, and FoldX score), while the columns divide the plots by structural annotation.

Additional Figure A1 highlights that AlphaMissense scores correlate with diverse structural and functional features across variant structural annotation categories. Variants in annotated regions such as interfaces, buried sites, and structural elements show progressively higher conservation, destabilising FoldX ΔΔG values, and elevated EVE scores with increasing AlphaMissense scores. These trends support the alignment of AlphaMissense predictions with biologically meaningful features. Notably, even variants lacking prior annotation (“none”) exhibit coherent score-feature relationships, underscoring the model’s potential to capture latent functional constraints. This supplementary analysis provides an additional layer of validation for the interpretability and relevance of AlphaMissense predictions.

RMSF and SASA metrics showed similar trends to each other, but in the opposite direction compared to the other descriptors—both decreased with increasing AlphaMissense scores. This concordance likely stems from the intrinsic relationship (rho = 0.6, CI [0.59, 0.63]) between solvent exposure and residue mobility (Table A2).

### 2.2. Missense Tolerance and Differential Stability in AGAL Variants

**Figure 2:**
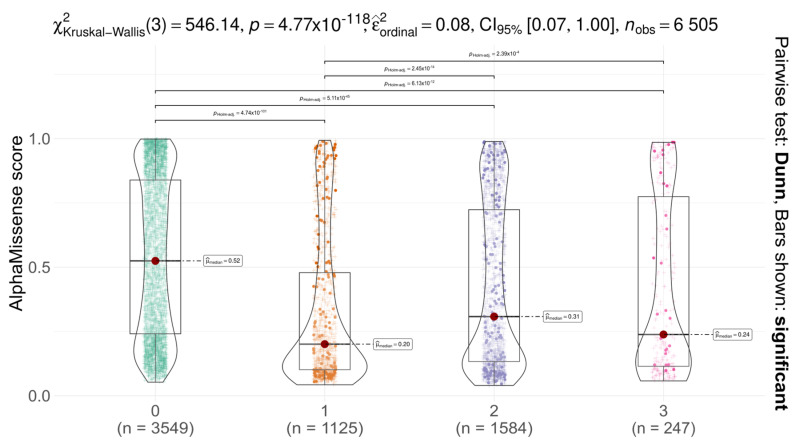
Boxplot of AlphaMissense scores stratified by codon position (0 = not an SNP; 1, 2, 3 means substitution in the first, second, and third nucleotides, respectively). Variants reported in ClinVar are shown as solid dots; unreported variants are indicated by “+” symbols. with the “0” group containing substitutions not possible via single nucleotide polymorphism (SNP). The plot title reports the Kruskal–Wallis test statistic and Dunn’s post hoc results.

We analysed the distribution of AlphaMissense scores for AGAL missense variants across different codon positions (first, second, third) and compared them with non-SNP substitutions (Figure 2). Notably, ClinVar does not report any non-SNP missense variants at codon position 0 for AGAL, resulting in the absence of data points in this layer. A significant difference in score distributions was observed among codon positions (Kruskal–Wallis test with Dunn’s post hoc correction, *p* < 0.05), suggesting that substitutions at different positions have varying functional impacts. In all cases, the median AlphaMissense score was below the pathogenicity threshold (0.564), and for all SNPs, scores remained below the uncertainty threshold (0.340), indicative of likely benign effects. These findings suggest that AGAL is relatively tolerant to missense variation, as predicted by AlphaMissense. Comparison with gnomAD constraint metrics provides a more nuanced view. The gene shows moderate constraint against missense variation (Z = 1.88; o/e = 0.58), indicating some evolutionary selection against amino acid substitutions, though not at the level seen in highly constrained genes (Z > 3) [23].

**Figure 3:**
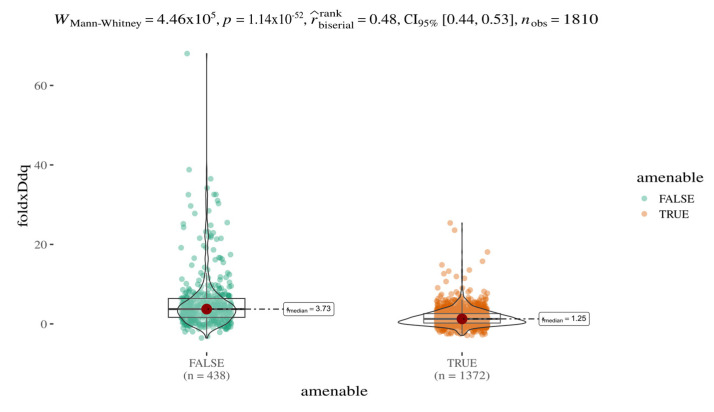
Stability of amenable and non-amenable variants. Boxplot of FoldX-predicted ΔΔG values stratified by amenability status (green: non-amenable; orange: amenable). The plot title reports test statistics from the Mann–Whitney U test used to assess differences between groups.

Systematic integration of multiple variant annotations (e.g., EVE and AlphaMissense scores) enables both a stratified analysis of variant pathogenicity classes (e.g., pathogenic vs. benign) and a comparative evaluation of additional features such as predicted protein stability. In this context, we investigated whether amenable and non-amenable variants [24] differ in terms of predicted stability changes (ΔΔG), as calculated by FoldX (Figure 3). A statistically significant and robust difference was observed between the two groups (Mann–Whitney U test, *p* < 1.14 × 10^−52^), with a median ΔΔG ratio of approximately 3:1. These results, based on a sample size of 1810 variants, suggest that amenable mutations are on average less destabilising; predicted protein destabilisation may contribute to the amenability profile of AGAL variants.

### 2.3. Agreement Between AlphaMissense and Alternative Predictors

To better understand how machine learning–based variant effect predictors integrate available biological information, we systematically compared AlphaMissense scores with three orthogonal metrics: (i) EVE scores, to assess whether pathogenicity predictions are more influenced by sequence conservation or structural context; (ii) FoldX-predicted ΔΔG values, to evaluate the potential contribution of variant-induced destabilisation; and (iii) RMSF (root-mean-square fluctuation) values derived from molecular dynamics simulations of the AGAL dimer, to test whether residue flexibility influences pathogenicity estimates. The first comparison highlights the added value of including structural information in pathogenicity prediction, while the second and third analyses provide indirect insights into the internal representations learnt by AlphaMissense. All analyses were restricted to SNP-derived missense variants (i.e., variants from codon positions 1–3), excluding the non-SNP class (“0”).

**Figure 4:**
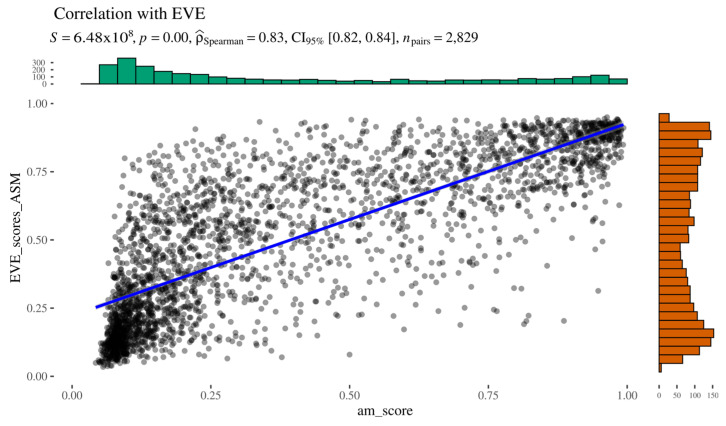
AlphaMissense to EVE relationship.

As indicated by Spearman’s rank correlation analysis, AlphaMissense and EVE scores show a strong positive correlation (ρ = 0.83), supported by a low *p*-value and a narrow CI (Figure 4). Overall, the two predictors demonstrate good concordance, particularly at the extremes of the pathogenicity spectrum, where both methods agree on highly likely pathogenic or benign variants. However, no clear pattern was observed among the outliers, who did not share common features. These results suggest that, at least in the context of AGAL, AlphaMissense and EVE produce comparable results.

**Figure 5:**
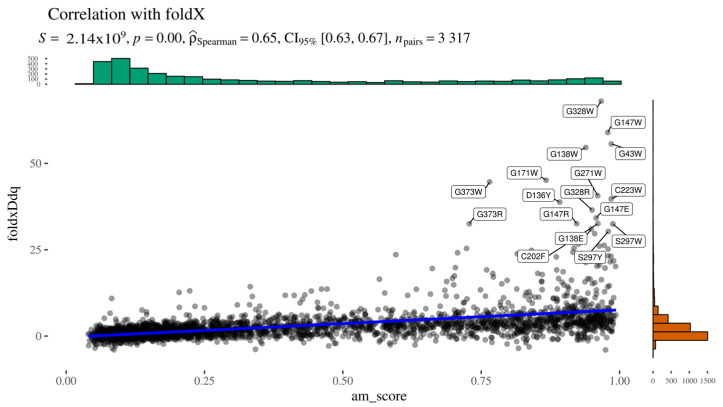
Alphamissense to FoldX relationship.

To indirectly assess whether AlphaMissense pathogenicity scores are influenced by predicted structural destabilisation, we computed the Spearman correlation between AlphaMissense scores and FoldX-predicted ΔΔG values (Figure 5). The two predictors showed a moderate positive correlation, with Spearman’s ρ = 0.65, a low *p*-value, and a narrow confidence interval, indicating statistical robustness. A closer inspection of the scatter plot reveals a subset of variants deviating markedly from the main trendline. This deviation likely arises from the bounded nature of AlphaMissense scores (limited to [0,1]), in contrast to the unbounded range of ΔΔG values. These outliers cluster in the upper-right quadrant of the graph, representing variants with both high predicted pathogenicity (AlphaMissense > 0.7) and substantial destabilisation (ΔΔG > 10 kcal/mol).

**Figure 6:**
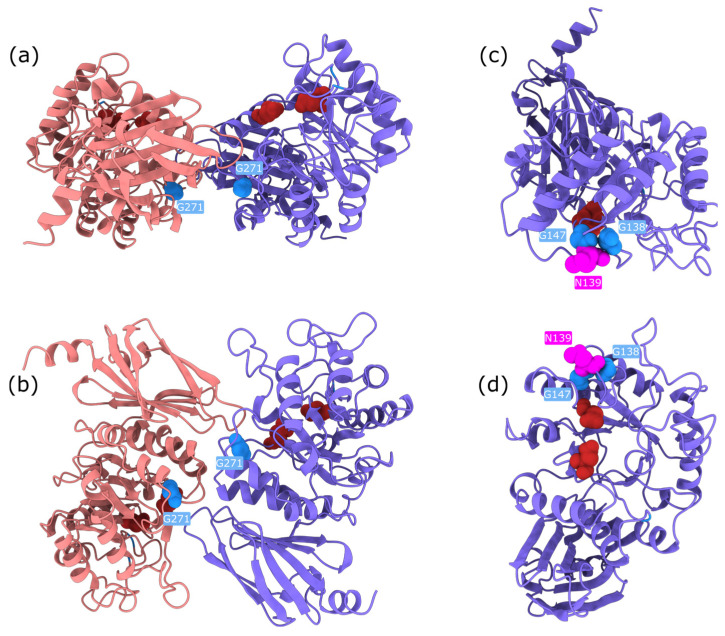
Structurally relevant glycine residues in AGAL, annotated on the AlphaFold3-predicted structure of human alpha-galactosidase (UniProt: P06280, AGAL_HUMAN). Catalytic active-site residues are shown in dark red. The two subunits of the homodimer are coloured salmon and slate blue. Panels (**a**,**c**) show top views; panels (**b**,**d**) show side views. Panels (**a**,**b**) highlight Gly271 (light blue, labelled), located near the dimerisation interface. Panels (**c**,**d**) show the structural context of Gly138 and Gly147 (dark blue), along with the N-linked glycosylation site at Asn139 (magenta, labelled).

Upon examination, these high-impact variants are predominantly buried glycine substitutions, with the exception of three notable residues: Gly271, Gly147, and Gly138 (Figure 6). Gly271 is located near the dimerisation interface, suggesting that its mutation could disrupt protein–protein interactions. Gly147 and Gly138 are adjacent to a known glycosylation site (Asn137); in particular, Gly138 may be critical for maintaining the consensus sequence required for proper glycosylation. The functional impact of Gly147 remains unclear, though its proximity to a post-translational modification site suggests potential relevance. These findings highlight specific residues as promising candidates for experimental validation to further investigate their role in AGAL function and pathology.

**Figure 7:**
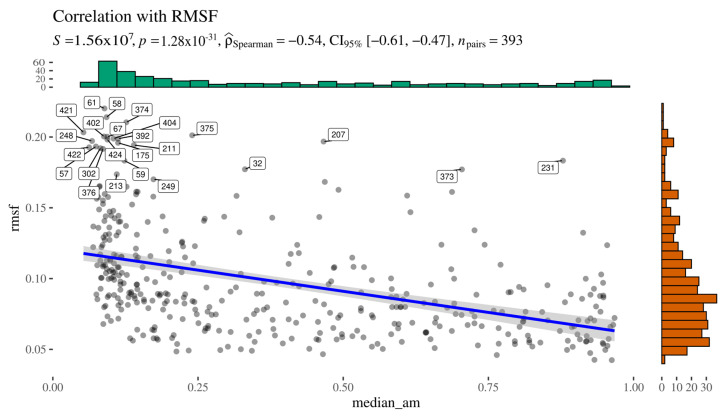
Alphamissense to RMSF relationship.

To investigate whether AlphaMissense predictions reflect residue-level flexibility—i.e., whether its internal representation captures aspects of molecular dynamics—we assessed the correlation between AlphaMissense scores and root-mean-square fluctuation (RMSF) values derived from molecular dynamics simulations of the AGAL homodimer (Figure 7). We excluded solvent-accessible surface area (SASA) from this analysis, as it is readily inferred from static structural models, and, importantly, AlphaFold and AlphaMissense are not explicitly provided with the notion of “surface.”

To avoid spurious correlations—particularly those potentially mediated by SASA—we first evaluated the relationship between RMSF and SASA. As expected, the two metrics were moderately correlated (Spearman’s ρ = 0.61), which complicates the interpretation of any observed correlation between AlphaMissense scores and RMSF. Specifically, it becomes difficult to disentangle whether any association reflects a genuine link between pathogenicity prediction and flexibility or whether it is an indirect effect mediated by residue exposure.

Nonetheless, this analysis provides a framework to explore the extent to which machine learning–based predictors might incorporate dynamic features, even when trained exclusively on static structural inputs.

### 2.4. In-Depth Examination of Exemplary Variants with Divergent Predictions

We selected SNPs in non-relevant positions (e.g., not in the catalytic/binding positions or at the dimerisation interface), which resulted in either pathogenic according to AlphaMissense or non-VUS in AlphaMissense and observed in ClinVar. We then filtered out all positions for which fewer than eight variants were observed: Arg100 and Arg112. We focused on structural evaluation of the latter, since it has been seen in association with Fabry patients (cross-referenced in ClinVar as VCV000092551.8, VCV000092550.27, and VCV000195028.71).

For residue Arg112, EVE assigns a high pathogenicity score, consistent with its strong evolutionary conservation. FoldX ΔΔG values for the Arg112Cys and Arg112His mutations are below 2 kcal/mol, indicating a destabilising effect. Additionally, the residue shows high RMSF and SASA values, suggesting structural flexibility and solvent exposure. In contrast, AlphaMissense assigns relatively low pathogenicity scores to these mutations.

**Figure 8:**
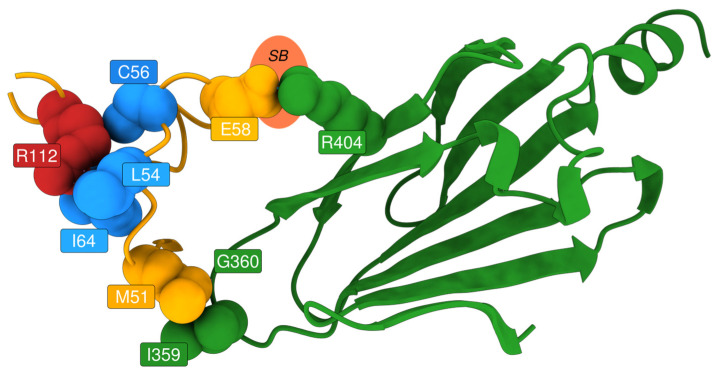
Detail of Arg112-centred intra- and inter-chain interactions on the AlphaFold3-predicted structure of human alpha-galactosidase (UniProt: P06280, AGAL_HUMAN). Residue Arg112 is highlighted in red, with its direct interaction partners shown in light blue. Residues on the adjacent loop, which is stabilised by Arg112, are coloured yellow. All residues coloured red, blue, yellow, and orange belong to one subunit of the AGAL homodimer, while the green structure represents the interacting subunit. A putative salt bridge (SB) between the subunits (Glu58 <-> Arg404) is indicated schematically.

Structural bioinformatics analysis allowed to resolve this discrepancy. ChimeraX visualisation revealed that Arg112 plays a key role in stabilising a loop near the dimer interface of AGAL. Mutations at this site disrupt the loop, potentially affecting inter-subunit interactions and leading to protein destabilisation (Figure 8). While AlphaMissense incorporates structural information, it lacks awareness of AGAL’s dimeric configuration, likely explaining its underestimation of pathogenicity. Experimental evidence further supports the destabilising nature of these mutations. Thus, EVE correctly identifies the functional importance of Arg112, while AlphaMissense misses critical structural context.

## 3. Materials and Methods

### 3.1. Data Collection

To explore and integrate relevant genetic information, we employed an analytical workflow developed in the R [25] programming language using the RStudio (v. 4.4.1) [26] integrated development environment (IDE). Data were retrieved from multiple sources, including public databases such as gnomAD [27] and HumSavar [28], as well as predictive models like EVE [21] and AlphaMissense [20]. All the scripts are available upon request.

Data extraction and wrangling were performed primarily using functions from the Tidyverse package [29]. The preprocessing phase included standardisation of amino acid codes (three-letter to one-letter), removal of incomplete or duplicated entries, and harmonisation of variant nomenclature into a unified format (reference amino acid—position—alternate amino acid). Following data cleaning, all sources were merged into a single, structured dataset, ensuring consistent alignment across variant representations and formats.

### 3.2. Data Curation and Subsetting

The final dataset underwent manual quality control to ensure consistency and cohesion across the various data sources. Each column from the different databases was systematically reviewed, and similar fields were identified and grouped to enable coherent and meaningful data integration. These groupings were critical for organising and summarising the information effectively.

Initially, we separated columns related to variant scoring, particularly those derived from AlphaMissense and EVE. A second set of groupings was performed for variant frequency data. Redundant frequency columns extracted from EVE were removed, as frequency information was already included during the construction of the primary dataset.

Further groupings were based on variant annotations, incorporating clinical significance data from sources such as ClinVar [30]. Following the integration process, a further curation step was applied to ensure that all mutation identifiers followed a standardised format: [aa-pos-aa], where “aa” represents the one-letter amino acid code and “pos” the residue position. We excluded mutations that did not conform to this format. This filtering step ensured the structural integrity of the dataset, allowing us to focus exclusively on mutations meeting our defined criteria and to enhance the consistency and reliability of downstream analyses.

### 3.3. Mutant Characterisation

To investigate AGAL mutations implicated in Fabry disease at the structural level, we adopted a computational approach utilising the ProtVar Application Programming Interface (API) [31], which provides structured data on protein variants.

A custom R function, named get_foldx, was developed using the httr, jsonlite, and tidyverse libraries. This function queries the ProtVar API by specifying the UniProt accession number for AGAL (P06280) and the exact residue position within the AGAL protein sequence (residues 1–429). The function constructs the API request URL by concatenating the accession and residue position, then performs an HTTP GET request via httr. The resulting JSON response is parsed and transformed into a structured R object using jsonlite.

We iterated over the entire residue range (1–429) using a for loop, systematically calling get_foldx to retrieve mutational data for each position. We used the same R environment and libraries to retrieve additional annotations from ProtVar by modifying query parameters.

The conservation data, provided by ProtVar, are derived from a bioinformatic pipeline that combines BLAST-based sequence alignments with ScoreCons [32,33], a tool for quantifying residue conservation across homologous proteins. These analyses offer insights into the structural and evolutionary constraints of each residue.

### 3.4. Molecular Dynamics

To generate a more complete AGAL dimer model for molecular dynamics simulations, we started from the AlphaFold-predicted structure AF-P06280-F1-model_v4. Using ChimeraX [34], we removed the signal peptide (residues 1 to 31) and superimposed two copies of the resulting structure onto the two chains of the X-ray crystallographic structure 3HG2. We selected this structure due to its absence of mutations, lack of bound ligands, and the highest resolution available under these conditions. For the second model, we introduced the L300F mutation using the “swapaa: 300 PHE criteria c” function in ChimeraX, generating the fewest number of clashes. Molecular dynamics simulations were performed using GROMACS [35] version 2022.3, with the OPLS-AA force field, TIP3P water model, and periodic boundary conditions. The simulation box was cubic, with a minimum distance of 2.0 nm between the protein and the box boundaries, and was neutralised using Na^+^ and Cl_−_ ions. To obtain a reliable model, we first performed 1 ns of energy minimisation using the steepest descent algorithm, followed by equilibration under isothermal–isovolumetric (NVT) and isothermal–isobaric (NPT) conditions for 500,000 steps each.

### 3.5. Structural Analysis

Structural analyses were performed using UCSF ChimeraX to assess solvent accessibility within the protein structures. The Solvent Accessible Surface Area (SASA) was calculated to evaluate the extent of solvent exposure of individual residues. SASA calculations were conducted in ChimeraX using a rolling probe algorithm that considers atomic radii and spatial arrangements. Root-mean-square fluctuation (RMSF) values were computed to estimate the average positional mobility of protein residues based on molecular dynamics simulations (see Section 3.4. Molecular dynamics).

### 3.6. Variant Annotation and Structural Data Integration

To comprehensively assess the structural and functional consequences of AGAL (P06280) mutations, we queried the ProtVar API to obtain data on protein stability and structural prediction quality. The retrieved data were organised into a dataframe structured to facilitate detailed analysis of protein variants. The data was annotated with structural properties based on inspection of the UniProt entry (P06280) except for the interface residues. For those, a consensus AGAL dimer interface was defined based on existing AGAL crystal structures, selecting residues interacting in at least 70% of subunits; this was further integrated with interface residues consistently predicted across the five AlphaFold 3 AGAL dimer models.

To characterise the dynamic and structural features of AGAL, we also computed root-mean-square fluctuation (RMSF) values based on the backbone atoms (N, Cα, C), which are stored in the dataset. These values provide insights into the flexibility of each residue in the mature protein. Similarly, solvent accessibility for each residue was measured using ChimeraX, and the resulting dataset contains per-residue SASA values. Notably, due to the cleavage of the signal peptide during maturation, both SASA and RMSF analyses start from residue 32. Due to its size and complexity, the final aggregated dataset was rendered as an interactive ShinyApp available at the following url: http://arfalas.shinyapps.io/shiny/ (accessed on November 2023).

### 3.7. Data Visualisation

Protein variant data was sourced from datasets pre-processed as described in previous sections. Data filtering and integration were performed using the dplyr and tidyr packages. Variants were retained if they involved a change between standard amino acids (i.e., single nucleotide polymorphisms, SNPs). The dataset was further enriched by joining with structural annotations and information on chaperone amenability. Variants with low solvent-accessible surface area (SASA ≤ 0.5) and no prior annotation were classified as “buried”. Clinical significance (CS) values were categorised into three groups: Benign, Pathogenic, and Variants of Uncertain Significance (VUS). AlphaMissense scores (am_score) were also discretised into the same qualitative classes using established threshold values.

To explore relationships between AlphaMissense scores and structural or functional features (e.g., RMSF, FoldX ΔΔG, and EVE scores), scatter plots were generated using ggstatsplot::ggscatterstats(), which applies non-parametric correlation analyses. Group comparisons of AlphaMissense scores based on codon changes and chaperone amenability were visualised using ggstatsplot::ggbetweenstats(), with statistical summaries included directly in the plots.

Faceted plots were employed to analyse score distributions across mutations and annotation categories. All visualisations were created using the ggplot2 and ggstatsplot packages [36].

## 4. Conclusions

The advent of artificial intelligence in biochemistry has profoundly transformed our approach to predicting pathogenic variants [37]. In particular, the development of AlphaMissense represents a significant leap forward, enabling pathogenicity predictions for every possible missense substitution across the human proteome [20]. This is achieved by leveraging deep learning models trained on both structural predictions from AlphaFold and population-level variant frequency data.

Using human AGAL—a protein characterised by thousands of known missense variants—as a case study, we explored the consistency between AI-based predictors such as AlphaMissense and EVE and structure-based parameters. Interestingly, we observed a strong concordance between AlphaMissense and EVE, while the relationship between AlphaMissense scores and structural dynamics proved more complex.

A notable aspect of AlphaMissense is its ability to generate predictions for variants that are not naturally occurring via single nucleotide polymorphisms (SNPs), which constitute the majority of its output. As expected, these variants are absent from databases like ClinVar, raising important questions about their biological relevance and interpretability.

Integrating AlphaMissense with tools like EVE and FoldX gives us a more comprehensive view of variant effects. Notably, AlphaMissense does not account for quaternary structure or protein–protein interfaces and thus may overlook the pathogenic potential of mutations affecting oligomerisation. In such contexts, structural analysis remains indispensable.

Finally, our investigation contributes to the ongoing discussion regarding the molecular basis of responsiveness to pharmacological chaperones such as DGJ. While active-site variants are anticipated to fail to bind the drug, the lack of response in specific non-catalytic missense variants remains an intriguing, unresolved phenomenon. Our data suggest that non-responsive variants are significantly less stable than responsive ones, offering a new perspective for understanding differential DGJ sensitivity and guiding future therapeutic strategies.

## Data Availability

The original contributions presented in this study are included in the article. Further enquiries can be directed to the corresponding author.

## Abbreviations

The following abbreviations are used in this manuscript:

AGAL	α-galactosidase A
ERT	enzyme replacement therapy
EVE	Evolutionary Model of Variant Effect
FD	Fabry disease
Gb3	globotriaosylceramide
PCT	pharmacological chaperon therapy
RMSF	root-mean-square fluctuation
SASA	solvent-accessible surface area

## Appendix A

**Table A1:** Spearman correlation coefficients between AlphaMissense and other metrics/properties. It is worth noting that *p*-value and confidence interval are not diagnostic for the analysed case (*n* = 8151).

Pair	Lower	Rho	Upper	Adj.p
am_score vs. EVE_scores_ASM	0.790	0.803	0.815	0
am_score vs. conservation.score	0.602	0.622	0.641	0
am_score vs. sasa	−0.637	−0.617	−0.596	0
am_score vs. rmsf	−0.488	−0.466	−0.443	0

**Table A2:** Spearman correlation coefficients between metrics/properties. For AlphaMissense correlations see Table A1. It is worth noting that *p*-value and confidence interval are not diagnostic for the analysed case (*n* = 8151).

Pair	Lower	Rho	Upper	Adj.p
EVE_scores_ASM vs. conservation.score	0.738	0.753	0.768	0
EVE_scores_ASM vs. sasa	−0.514	−0.490	−0.466	0
EVE_scores_ASM vs. rmsf	−0.460	−0.441	−0.422	3.59 × 10^−321^
EVE_scores_ASM vs. foldxDdq	0.517	0.541	0.564	0
conservation.score vs. sasa	−0.518	−0.495	−0.471	0
conservation.score vs. rmsf	−0.490	−0.467	−0.444	0
conservation.score vs. foldxDdq	0.432	0.452	0.472	0
sasa vs. rmsf	0.585	0.606	0.626	0
sasa vs. foldxDdq	−0.588	−0.566	−0.544	0
rmsf vs. foldxDdq	−0.474	−0.453	−0.430	0
